# The covid-19 pandemic and hospital morbidity due to mental and behavioral disorders in Brazil: an interrupted time series analysis, from January 2008 to July 2021

**DOI:** 10.1590/S2237-96222023000100016

**Published:** 2023-04-14

**Authors:** Carolina Novaes Carvalho, Sandra Fortes, André Peres Barbosa de Castro, Juan Cortez-Escalante, Thiago Augusto Hernandes Rocha

**Affiliations:** 1 Organização Pan-Americana da Saúde, Unidade Técnica de Doenças Transmissíveis e Determinantes Ambientais da Saúde, Brasília, DF, Brazil; 2 Universidade do Estado do Rio de Janeiro, Faculdade de Ciências Médicas, Rio de Janeiro, RJ, Brazil; 3 Ministério da Saúde, Secretaria de Vigilância em Saúde, Brasília, DF, Brazil; 4 Organização Pan-Americana da Saúde, Unidade Técnica de Vigilância, Preparação e Resposta a Emergências e Desastres, Brasília, DF, Brazil; 5 Duke University, Duke Global Health Institute, Durham, North Carolina, United States of America

**Keywords:** Covid-19, Mental Disorders, Hospitalization, Hospital Information Systems, Mental Health, Interrupted Time Series, Covid-19, Trastornos mentales, Hospitalización, Sistemas de Información en Hospital, Salud Mental, Series de Tiempo Interrumpido, Covid-19, Transtornos Mentais, Hospitalização, Sistemas de Informação Hospitalar, Saúde Mental, Série Temporal Interrompida

## Abstract

**Objective:**

to analyze records of hospitalizations due to mental and behavioral disorders before and after the beginning of the covid-19 pandemic in Brazil, from January 2008 to July 2021.

**Methods:**

this was a descriptive ecological interrupted time series study, using secondary data retrieved from the Brazilian National Health System Hospital Information System; a time series analysis of hospitalizations was conducted based on a population-weighted Poisson regression model; relative risk (RR) and respective 95% confidence intervals (95%CI) were calculated.

**Results:**

we identified 6,329,088 hospitalizations due to mental and behavioral disorders; hospitalization rates showed an 8% decrease (RR = 0.92; 95%CI 0.91;0.92) after the start of the pandemic, compared to the pre-pandemic period.

**Conclusion:**

the pandemic changed the trend of hospitalizations due to mental and behavioral disorders in Brazil; the drop observed in the period is evidence that the pandemic affected the mental health care network.

## Resumo

**Objetivo:**

analisar as internações por transtorno mental e comportamental, antes e após o início da pandemia de covid-19 no Brasil, de janeiro de 2008 a julho de 2021.

**Métodos:**

estudo ecológico descritivo de série temporal interrompida, com dados registrados no Sistema de Informações Hospitalares do Sistema Único de Saúde (SIH/SUS); realizada análise da série temporal das internações baseada em um modelo de regressão de Poisson, ponderado pela população; calculado o risco relativo (RR), com intervalo de confiança de 95% (IC_95%_).

**Resultados:**

foram identificadas 6.329.088 internações por transtornos mentais e comportamentais; as taxas de internação apresentaram um decréscimo de 8% (RR = 0,92; IC_95%_ 0,91;0,92) após o início da pandemia, em relação ao período pré-pandemia.

**Conclusão:**

a pandemia modificou a tendência das internações por transtornos mentais e comportamentais no Brasil; a queda observada no período é evidência de que a pandemia afetou a cadeia de cuidado estruturada para saúde mental.

## Resumen

**Objetivo:**

analizar las hospitalizaciones por trastornos mentales y del comportamiento antes y después del inicio de la pandemia de covid-19 en Brasil, desde enero 2008 hasta julio 2021.

**Método:**

estudio ecológico descriptivo de series temporales interrumpidas, con datos registrados en el Sistema de Informações Hospitalares del Sistema Único de Saúde; se realizó un análisis de series temporales de hospitalizaciones basado en modelo de regresión de Poisson, ponderado por la población; calculado el riesgo relativo (RR), con intervalo de confianza del (IC_95%_).

**Resultados:**

se identificaron 6.329.088 hospitalizaciones por trastornos mentales y del comportamiento; las tasas de hospitalización mostraron disminución del 8% (RR = 0,92; IC_95%_ 0,91;0,92) tras el inicio de la pandemia, en relación con el periodo prepandémico.

**Conclusión:**

la pandemia cambió la tendencia de hospitalizaciones por trastornos mentales y del comportamiento en Brasil; la caída observada en el período evidencia que la pandemia afectó la cadena asistencial estructurada para la salud mental.

**Table:** 

Study contributions	
**Main results**	A sharp decrease in hospitalization rates due to mental and behavioral disorders was seen in Brazil after the onset of the COVID-19 pandemic, this being evidence that the pandemic affected the mental health care network.
**Implications for services**	There is evidence that there is a delay between the appearance of the first symptoms of mental disorders and seeking specialized care, which means that the consequences of the pandemic for psychosocial care will probably be seen in the years to come.
**Perspectives**	The change in the National Mental Health Policy, in the period analyzed, may lead to a misinterpretation of the change in the pattern of hospitalizations due to the pandemic, requiring future studies in order to gain a better understanding of the phenomenon.

## INTRODUCTION

According to the International Health Regulations (IHR), the covid-19 pandemic is an international public health emergency, the highest level of alert issued by the World Health Organization (WHO).^1^ As at October 2022, there were more than 619 million confirmed cases worldwide and 6.5 million deaths had been recorded.^2^ In Brazil, there were more than 34 million confirmed cases, and more than 686,000 deaths caused by the disease.^2^ ^),(^ ^3^

Due to its potential for contagion, the characteristics of its evolution and the complexity of its management, covid-19 has posed challenges for health systems in the countries where it has spread.^4^ covid-19 mortality has been shown to be higher than that observed for seasonal influenza, and the complications caused by the virus lead to an increase in the demand for care, generating overload for all levels of care, especially tertiary care (hospital, intensive care). This behavior shown by covid-19 triggers public health crises, both in developing countries and in wealthy countries on all continents, creating a situation unprecedented in recent decades.^5^

 Clinical management of people with a highly contagious severe disease, with a little known clinical picture, and with no available treatment, had repercussions on the regular process of providing care related to the treatment and prevention of other diseases not associated with covid-19, as well as mental health. ^(^ ^6^ ^),(^ ^7^

Worldwide, the covid-19 pandemic has had impacts on the population’s mental health and psychosocial well-being. Psychological suffering, which affected different segments of the population, has been a consequence of the immediate effects of the virus on people’s health and measures to contain transmission, including social isolation, suspension of services and the resulting economic crisis.^8^ The WHO estimates that 30% to 50% of people have some psychological distress or have developed a mental health problem due to the pandemic. ^(^ ^9^

Fear, stress, feelings of helplessness, loneliness, insomnia, anger, depression, anxiety, post-traumatic stress, suicide ideations, suicide attempts and/or suicide itself are some of the manifestations reported in the literature.^10^ ^),(^ ^11^ These conditions can be seen in particular among people in isolation, as they accentuate psychological distress.^11^ Notwithstanding, people who had mental disorders before the pandemic are among the groups most vulnerable to the worsening of mental health problems.^12^

Mental health care within the scope of the Brazilian National Health System (Sistema Único de Saúde - SUS) aims to ensure that people with mental suffering or mental disorders and with needs resulting from the use of crack, alcohol and other drugs have access to comprehensive and humanized care.^15^ The Psychosocial Care Network (Rede de Atenção Psicossocial - RAPS) was structured to meet this purpose. The RAPS brings together different services in a territorialized network, and includes everything from primary health care to hospital care, reinforcing its articulation as a way of guaranteeing the effectiveness of care.^16^

The RAPS Hospital Care component offers support in general, pediatric and maternity hospitals, through short-term hospitalizations, to people with mental suffering or disorders, jointly with the Psychosocial Care Centers (Centros de Atenção Psicossocial - CAPS) and other health and social service points of care.^17^

Studies evaluating the secondary effects of covid-19 on mental health are scarce, despite their importance in the current scenario. The population, in general, when exposed to the risk of contamination and the possibility of becoming ill due to covid-19, can experience situations of vulnerability that enhance the development of mental health problems.^14^ This study is part of this context and aimed to analyze the trend of hospitalizations due to mental and behavioral disorders before and after the beginning of the covid-19 pandemic in Brazil.

## METHODS

*Study design*

This was a descriptive ecological interrupted time series study, using secondary data retrieved from the Brazilian National Health System Hospital Information System (Sistema de Informações Hospitalares do Sistema Único de Saúde - SIH/SUS). We analyzed hospitalizations due to mental and behavioral disorders from January 2008 to July 2021. 

*Setting*

According to Demographic Census data, in 2010 Brazil had a population of 190,755,799 inhabitants,^18^ distributed over five geopolitical regions - North, Northeast, Midwest, Southeast and South - each of which have different demographic, economic and social characteristics. 

As at December 2020, Brazil had 1,927 beds in Reference Hospital Services for Mental Health, Alcohol and other Drugs. In 2012, these services were regulated and intended to care for clinical comorbidities resulting from the use of psychoactive substances, especially abstinence and severe intoxication, as well as management of mental health crisis situations, together with the CAPS and other RAPS points of care. With regard to beds in psychiatric hospitals, between 2002 and 2020, nationwide the SUS reduced its psychiatry beds by 37,464, in line with the recommendations of the Psychiatric Reform Law (Law No. 10216/2001), intended to reorient the mental health care model in Brazil.^19^

This study examined records of hospitalizations associated with mental and behavioral disorders, as per the International Statistical Classification of Diseases and Related Health Problems - 10^th^ Revision (ICD-10). We conducted a comparative analysis, taking two moments in time: a period prior to the covid-19 pandemic and a period following the onset of the pandemic.

We took January 2008 to February 2020 as the pre-covid-19 pandemic period, and April 2020 to July 2021 as the post-onset period. The intervention measure considered was the WHO declaration of the covid-19 pandemic, in March 2020.

*Participants and variables*

For the purposes of this study, mental and behavioral disorders were based on the records of hospitalizations of people resident in Brazil, from January 2008 to July 2021, with primary diagnosis classified as follows: mental and behavioral disorders (F00-F99), symptoms and signs involving emotional state (R45) and external causes of morbidity and mortality (X60-X84), as per ICD-10 chapters V, XVIII and XX, respectively. Given that the data were available until July 2021 and not for the whole of 2021, the time series was structured month by month in order to enable comparison.

The following variables were included in the study:

- health service user identification: date of birth, race/skin color (White, Black, mixed race, Asian, Indigenous, unknown and not informed), sex (male, female), region of residence (Midwest, Northeast, North, Southeast, South and not informed);
- main ICD-10 code (F00-F99, R45 and X60-X84);
- nature of hospitalization (elective, urgency, occupational accident, accident on the way to or from work, other types of transport accident, other types of injury and poisoning due to chemical or physical agents);
- hospitalization date;
- number of inpatient days;
- number of charged days;
- total hospitalization cost (in BRL);
- reason for charging (grouped into the following categories: discharge, inpatient stay, transfer, death, administrative case closure and not informed); and
- occurrence of death in the period (no, yes).
*Data sources/measuring*

Anonymous SIH/SUS databases were used as sources of information. We chose SIH/SUS databases due to the public availability of data on hospitalizations paid for by SUS. The SUS user population corresponds to 75% of the Brazilian population and, due to its scope, the SIH/SUS allows for analysis of changes in trends linked to mental health hospitalizations. The data were extracted in October 2021 from the SUS Department of Information Technology (DATASUS) website, and hospitalizations were aggregated by region of residence of people with mental suffering or disorder and year of hospitalization. 

The third digit of the ICD-10 code of the primary diagnostic field was excluded, and all subdivisions of groups F00-F99, R45 and X60-X84 were considered. The ages of people with mental and behavioral disorders were calculated by the difference between the date of hospital admission and date of birth. 

Population data from the 2010 Demographic Census and intercensal population projections, made available by the Brazilian Institute of Geography and Statistics (Instituto Brasileiro de Geografia e Estatística - IBGE), were used to estimate hospitalization rates per resident population^20^ multiplied by 10,000 inhabitants, for Brazil as a whole.

*Statistical analysis*

The continuous variables were described using mean values and standard deviation (SD); while the categorical variables were described using frequencies and percentages. 

In order to identify changes in the level and slope of trends in hospitalization rates for the pre-pandemic and post-pandemic onset periods, we opted to use interrupted time series analysis. 

The time series analysis of mental and behavioral disorder hospitalizations was based on a population-weighted Poisson regression model. Mental and behavioral hospitalizations per year were used to measure the outcome. Changes in the hospitalization rate over to time and due to the pandemic were analyzed. The pandemic variable was added to the model as a dummy. We calculated relative risk (RR) taking a 95% confidence interval (95%CI) and taking models with a p-value < 0.05 to be statistically significant. 

Number of hospitalizations ~ log (population) + pandemic + time + weighted harmonics 

 The selection of this model was based on estimated count data - in this case, the number of hospitalizations. Thus, the count data was modeled directly, using the population (transformed log) as a compensation variable. Residual autocorrelation parameters were used to validate the results. The choice of the method shown above was based on the recommendations made by Bernal et al. (2017).^21^

The analyses were performed using the R statistical language, both for data analysis and for graph production, with the support of the lmtest, EPI and tsModel packages.

We used anonymous secondary public database information. As such, the study was exempted from submission to a Research Ethics Committee. 

## RESULTS

We analyzed 6,329,088 records of hospitalizations due to mental and behavioral disorders. The region of the country with the highest number of hospitalizations was the Southeast (3,130,919; 49.0%), while the Northern region had the lowest number (113,254; 1.8%) (Table 1).

**Table 1:** Distribution of mental and behavioral disorder hospitalizations by year and geographic region, Brazil, January 2008 to July 2021

Year of hospitalization	Regions of residence						
	Midwest	Northeast	North	Southeast	South	Not informed	Total
	n (%)	n (%)	n (%)	n (%)	n (%)	n (%)	n
2008	33,553 (5.5)	132,381 (22.0)	7,567 (1.2)	331,153 (55.0)	99,824 (16.0)	884 (0.1)	605,362
2009	34,272 (5.8)	129,571 (22.0)	7,966 (1.4)	311,595 (53.0)	103,360 (18.0)	736 (0.1)	587,500
2010	34,700 (6.0)	125,247 (22.0)	8,929 (1.5)	302,688 (52.0)	109,164 (19.0)	1,278 (0.2)	582,006
2011	34,439 (6.0)	116,187 (20.0)	9,131 (1.6)	302,721 (52.0)	111,723 (19.0)	2,956 (0.5)	577,157
2012	34,449 (6.3)	107,321 (20.0)	8,757 (1.6)	285,330 (52.0)	108,998 (20.0)	3,338 (0.6)	548,193
2013	31,081 (6.2)	96,496 (19.0)	7,764 (1.5)	261,447 (52.0)	103,917 (21.0)	2,942 (0.6)	503,647
2014	26,123 (5.6)	87,466 (19.0)	8,254 (1.8)	241,232 (52.0)	101,075 (22.0)	3,024 (0.6)	467,174
2015	25,643 (6.1)	80,170 (19.0)	7,722 (1.8)	209,517 (50.0)	96,244 (23.0)	3,542 (0.8)	422,838
2016	21,586 (5.7)	74,337 (20.0)	7,770 (2.0)	179,561 (47.0)	92,666 (24.0)	3,291 (0.9)	379,211
2017	20,070 (5.6)	70,535 (20.0)	7,715 (2.2)	160,001 (45.0)	95,733 (27.0)	3,548 (1.0)	357,602
2018	24,299 (6.9)	70,110 (20.0)	8,291 (2.4)	150,400 (43.0)	98,558 (28.0)	-	351,658
2019	25,934 (7.3)	70,720 (20.0)	7,568 (2.1)	152,513 (43.0)	97,156 (27.0)	-	353,891
2020	23,683 (7.8)	62,192 (21.0)	7,660 (2.5)	126,606 (42.0)	81,662 (27.0)	-	301,803
2021	23,127 (7.9)	62,795 (22.0)	8,160 (2.8)	116,155 (40.0)	80,809 (28.0)	-	291,046
Total	392,959 (6.2)	1,285,528 (20.0)	113,254 (1.8)	3,130,919 (49.0)	1,380,889 (22.0)	25,539 (0.4)	6,329,088

Males accounted for 64.0% of hospitalizations; 30.6% were of White race/skin color, and 20.3% were of mixed race. Mean age was 42 years (SD = 15). The majority of hospitalizations were urgent (53.6%) and lasted, on average, 20 days (SD = 12), corresponding to 19 days - length of hospital stay used to calculate the amount to be paid by the SUS. The annual mean cost of hospitalization ​​was R$ 927 million (SD = 614). Of the total records, 48.2% of service users were discharged, 47.5% remained hospitalized and 2.6% were transferred to another establishment. Death accounted for 0.3% of hospitalizations. Primary diagnosis indicates that schizophrenia, schizotypal and delusional disorders accounted for 43.0% of hospitalizations, followed by mood (affective) disorders and mental and behavioral disorders due to alcohol use (14.0% each). Mental and behavioral disorders due to use of other psychoactive substances accounted for 13.0% of hospitalizations (Table 2).

**Table 2:** Characterization of mental and behavioral disorder hospitalizations, Brazil, January 2008 to July 2021

Variable	n (%)
Sex	
Male	4,051,133 (64.0)
Female	2,277,955 (36.0)
Race/skin color	
White	1,939,843 (30.6)
Black	357,621 (5.7)
Mixed race	1,284,649 (20.3)
Yellow	17,892 (0.3)
Indigenous	3,045 (< 0.1)
Unknown	1,446,687(22.9)
Not informed	1,279,351 (20.21)
Nature of hospitalization	
Elective	1,278,903 (20.2)
Urgency	3,393,570 (53.6)
Other types of transport accident	569 (< 0.1)
Other types of injury and poisoning due to chemical or physical agents	46 (< 0.1)
Not informed	1,656,000 (26.2)
Reason for charging	
Discharge	3,051,625 (48.2)
Inpatient stay	3,008,571 (47.5)
Transfer to another establishment	164,794 (2.6)
Death	16,270 (0.3)
Administrative case closure	87,712 (1.4)
Not informed	116 (< 0.1)
Death during hospitalization	
No	6,312,818 (99.7)
Yes	16,270 (0.3)
Primary diagnosis associated with hospitalization, according to ICD-10a group and subgroup^a^	
Mental and behavioral disorders (F00-F99)	6,325,995 (99.1)
Dementia (F00-F03)	113,964 (1.8)
Mental and behavioral disorders due to use of alcohol (F10)	882,628 (14.0)
Mental and behavioral disorders due to use of other psychoactive substances (F11-F19)	811,596 (13.0)
Schizophrenia, schizotypal and delusional disorders (F20-F29)	2,722,422 (43.0)
Mood (affective) disorders (F30-F39)	910,998 (14.0)
Mental retardation (F70-F79)	335,135 (5.3)
Other mental and behavioral disorders (F04-F09, F50-F69, F80-F99)	507,687 (8.0)
Symptoms and signs involving emotional state (R45)	2,037 (< 0.1)
Intentional self-harm (X60-X84)	1,056 (< 0.1)

a) International Statistical Classification of Diseases and Related Health Problems, 10^th^ Revision.

**Figure 1:**
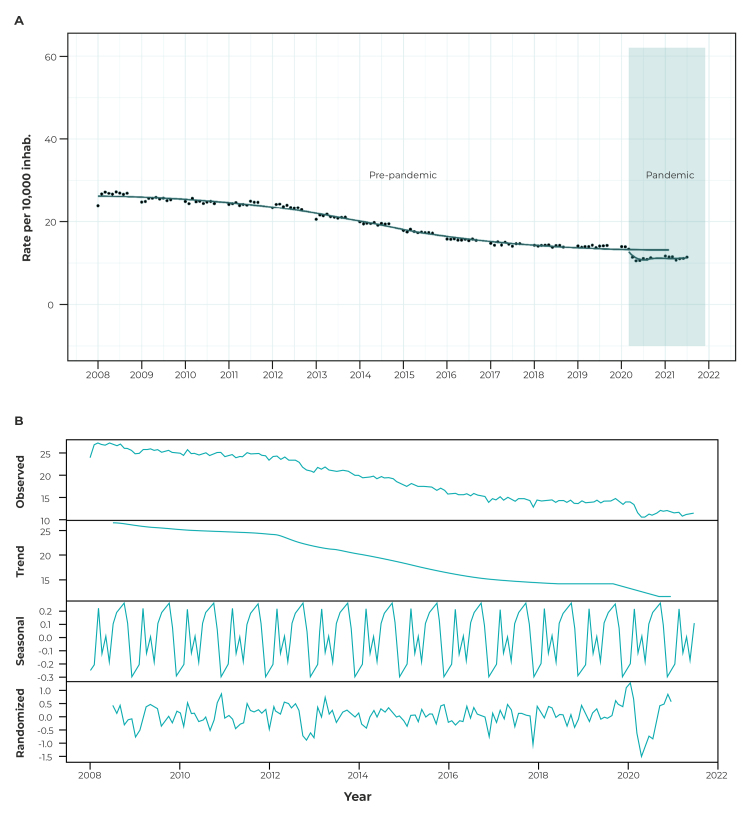
Mental and behavioral disorder hospitalization rate (A) and breakdown of the time series of mental and behavioral disorder hospitalization rates (B), before and after the onset of the COVID-19 pandemic, Brazil, January 2008 to July 2021

Figure 1 A shows the trend in hospitalization rates for mental and behavioral disorders per 10,000 inhabitants. The breakdown of the time series trend revealed a decrease (Figure 1B). As such, there was a downward trend in hospitalizations due to mental and behavioral disorders with effect from 2008 and throughout the pre-pandemic period, ranging from 27.3 to 12.8, with a certain stability between 2017 and 2020. 

Table 3 presents a summary of the analyses of hospitalizations before and after the start of the covid-19 pandemic in Brazil, from January 2008 to July 2021. We analyzed 148 months in the period before the pandemic and the WHO intervention, as well as 16 months in the period after the onset of the pandemic.

**Table 3:** Characterization of hospitalizations before and after the onset of the COVID-19 pandemic, Brazil, January 2008 to July 2021

Period	Variable	N	Mean hospitalizations	Standard deviation	Median	IQR^a^	Min.	Max.
Pre-pandemic^b^	Hospitalization	148	39,487.6	8,364.2	39,653.5	17,717.2	24,261.0	52,052.0
Pre-pandemic^b^	Rate	148	19.8	4.7	19.7	9.8	11.5	27.2
Post-onset of pandemic^c^	Hospitalization	16	24,059.2	1,500.2	23,822.0	1,522.5	22,282.0	28,237.0
Post-onset of pandemic^c^	Rate	16	11.3	0.7	11.2	0.7	10.5	13.3

a) Interquartile range; b) January 2008 to February 2021; c) April 2020 to July 2021.

Our analysis of the means and SD for the periods showed that there was a decrease in hospitalizations. In the pre-pandemic period, the mean number of hospitalizations was 39,487.6 (SD = 8,364.2), while after the onset of the pandemic, the mean number of hospitalizations fell to 24,059.2 (SD = 1,500.2). The interquartile range revealed lower data dispersion in the post-pandemic period.

Poisson regression analysis showed a statistically significant reduction in hospitalization rates in the period after the start of the pandemic, RR = 0.92 (95%CI 0.91;0.92), that is, an 8% decrease in hospitalizations due to mental and behavioral disorders associated with the pandemic period compared to the pre-pandemic period (Table 4). This result was statistically significant (p-value < 0.001), even when controlled for seasonality and stationarity effects.

**Table 4:** Relative risk (RR) and respective 95% confidence intervals (95%IC) of mental and behavioral disorder hospitalizations in the period following the onset of the pandemic, Brazil, January 2008 to July 2021

Characteristic	RR^a^	95%CI^b^	p-value
Pandemic	0.92	0.91;0.92	< 0.001
Time	0.99	0.99;0.99	< 0.001
Harmonic analysis (by month, with two sine and cosine pairs for the 12-month period)			
Harmonics 1	0.99	0.99;0.99	< 0.001
Harmonics 2	0.99	0.99;0.99	< 0.001
Harmonics 3	0.99	0.99;0.99	< 0.001
Harmonics 4	0.99	0.99;0.99	< 0.001

a) RR: Relative risk; b) 95%CI: 95% confidence interval.

## DISCUSSION

The results presented demonstrate a decrease in hospitalization rates due to mental and behavioral disorders in Brazil with effect from the onset of the covid-19 pandemic, and indicate that this decrease became more pronounced in the pandemic period covered by this study. This downward trend had become more visible since 2014, which may be related to the implementation of RAPS in the years prior to this.^22^

The inclusion of Specialized Psychiatric Hospitals as part of the RAPS, and respective alteration of the Hospital Admission Authorization (Autorização de Internação Hospitalar - AIH), as well as the incorporation of Specialized Reference Units in General Hospitals, which must have 80.0% of these beds occupied, can be considered elements that led to the stability of the drop in hospitalizations in the period from 2017 to the pre-pandemic in 2020. This may imply a change in the logic of hospitalization for mental health problems, reversing efforts made during more than ten years intended to deinstitutionalize health service users with mental and behavioral disorders.

The reduction in hospitalization rates found by the study for the pandemic period is in line with a recent report published by the WHO which highlighted that essential mental health services were interrupted in 93.0% of countries around the world during the period of social isolation,^23^ which may have led to a significant decrease in hospitalizations. On the other hand, in Brazil, studies indicate that, due to the high demand for intensive care beds (ICU) for people diagnosed with covid-19, some beds intended for service users with mental disorders, and even psychiatric hospitals, were transformed into covid-19 wards and intensive care beds.^24^ The sharp drop observed in the period is evidence that the pandemic has affected the mental health care network. The findings of the study point to a significant reduction in the volume of hospitalizations. 

In keeping with the most frequent results of studies conducted in Brazil,^16^ ^), (^ ^25^ ^)-(^ ^26^ hospitalizations of men were more frequent than those of women in the period analyzed. The profile of male psychiatric hospitalizations is more related to the use of psychoactive substances, while women are hospitalized due to behavior-related disorders.^27^ ^),(^ ^28^ A more specific analysis of this variable may point to more evidence regarding the maintenance or change in this pattern in the context of the pandemic.

Mean length of hospital stay was 20 days (DP = 12 days), in line with other findings in the Brazil.^28^ The mean hospital stay of 20 days can be interpreted positively, and may be associated with the community care model being prioritized to the detriment of the hospital-centered model, in keeping with the perspective advocated by the Psychiatric Reform.

Regarding hospitalizations according to ICD-10 subgroup, we found that the highest frequency of hospitalizations occurred due to schizotypal and delusional disorders, followed by mood (affective) disorders, mental and behavioral disorders due to alcohol use and mental and behavioral disorders due to use of other psychoactive substances, corroborating findings of studies conducted in Brazilian states.^25^ A report published in 2018 by the Pan American Health Organization (PAHO/WHO) on the burden of mental disorders in the Americas region, shows Brazil as having the highest percentage of disability due to mental disorders (36.5%). Depressive disorders and anxiety disorders accounted for 9.3% and 7.5%, respectively. Schizophrenia, considered to be a serious mental disorder, accounted for 1.6% compared to the other countries of the region.^29^

Studies indicate that the covid-19 pandemic caused significant changes in hospitalizations due to various causes, and this is one of the pioneering studies that analyzed data from Brazil, using quasi-experimental methods. Although access to health services has been affected in several medical specialties, it is important to consider that mental health is a historically neglected area and that the number of health services is insufficient. Evidence shows that there is a delay between the appearance of the first symptoms of a mental disorder and seeking specialist care, which means that we are likely to see the consequences for mental health in the coming years.^10^ The findings indicate that the pandemic significantly reduced mental health hospitalizations, and additional studies are needed to better understand the consequences of this finding. Additionally, it is known that the pandemic did not uniformly affect the different Brazilian states. As such, complementary studies are essential to understand local specificities.

Standing out among the limitations of this study is the change in the National Mental Health Policy during the period analyzed, which can lead to the misinterpretation that the pandemic has modified the pattern of hospitalizations, resulting in a decrease, whereas this effect may have resulted from the change in the logic of mental health care in favor of deinstitutionalization (dehospitalization), avoiding unnecessary or inappropriate hospitalizations, focusing instead on community-based treatment. However, the findings of this study are initial, and future studies should be developed in order to gain a better understanding of the phenomenon. In addition, analysis by region and/or state, to assess whether these trends are general or different across states, may be an important contribution to the topic.
